# Differences and similarities in clinical and functional responses among patients receiving tofacitinib monotherapy, tofacitinib plus methotrexate, and adalimumab plus methotrexate: a post hoc analysis of data from ORAL Strategy

**DOI:** 10.1186/s13075-021-02591-y

**Published:** 2021-08-24

**Authors:** Tsutomu Takeuchi, Roy Fleischmann, Noriko Iikuni, Harry Shi, Koshika Soma, Jerome Paulissen, Tomohiro Hirose, Josef S. Smolen

**Affiliations:** 1 Division of Rheumatology, Department of Internal Medicine, Keio University, Tokyo, Japan; 2 Rheumatology Division, Metroplex Clinical Research Center and University of Texas Southwestern Medical Center, Dallas, TX USA; 3 Pfizer Inc, New York, NY USA; 4 Pfizer Inc, Groton, CT USA; 5 Pfizer Japan Inc, Shinjuku Bunka Quint Building 22F, 3-22-7, Yoyogi, Shibuya-ku, Tokyo 151-8589 Japan; 6 Division of Rheumatology and Department of Medicine 3, Medical University of Vienna, Vienna, Austria

**Keywords:** Adalimumab, Methotrexate, Rheumatoid arthritis, Tofacitinib

## Abstract

**Background:**

This post hoc analysis assessed clinical and functional responses to tofacitinib monotherapy, tofacitinib + methotrexate (MTX), and adalimumab + MTX, in patients with rheumatoid arthritis enrolled in the ORAL Strategy study, including evaluation of patient-level data using cumulative probability plots.

**Methods:**

In the 12-month, phase IIIb/IV ORAL Strategy study, patients with rheumatoid arthritis and an inadequate response to MTX were randomized to receive tofacitinib 5 mg twice daily (BID), tofacitinib 5 mg BID + MTX, or adalimumab 40 mg every other week + MTX. In this post hoc analysis, cumulative probability plots were generated for mean percent change from baseline (%∆) in the Clinical Disease Activity Index (CDAI; clinical response) and mean change from baseline (∆) in the Health Assessment Questionnaire-Disability Index (HAQ-DI; functional response) at month 12. Median C-reactive protein (CRP) levels by time period were summarized by CDAI remission (≤ 2.8) status at months 6 and 12.

**Results:**

Data for 1146 patients were analyzed. At month 12, cumulative probability plots for %∆CDAI and ∆HAQ-DI were similar across treatments in patients with greater response. At lower levels of response, patients receiving tofacitinib monotherapy did not respond as well as those receiving combination therapies. With tofacitinib + MTX, numerically higher baseline CRP levels and numerically larger post-baseline CRP reductions were seen in patients achieving CDAI remission at months 6 and 12 vs those who did not.

**Conclusions:**

These results suggest that patients with a greater response did well, irrespective of which therapy they received. Patients with lesser response had better outcomes with combination therapies vs tofacitinib monotherapy, suggesting they benefitted from MTX. High pre-treatment CRP levels may be associated with better response to tofacitinib + MTX.

**Trial registration:**

ClinicalTrials.gov, NCT02187055. Registered on 08 July 2014.

**Supplementary Information:**

The online version contains supplementary material available at 10.1186/s13075-021-02591-y.

## Background

Rheumatoid arthritis (RA) is a chronic inflammatory disease affecting approximately 0.24% of the global population [1] and can lead to joint destruction, functional decline, disability, and decreased quality of life [2, 3]. An international, rheumatology-focused task force recommends that treatment should focus on controlling joint damage and normalizing function by abating disease activity [4]. The American College of Rheumatology (ACR) and European Alliance of Associations for Rheumatology (EULAR) recommend using a treat-to-target approach with the aim of achieving remission or, at least, low disease activity (LDA) [5, 6], to prevent progression of joint damage and optimize physical functioning [4].

Per ACR and EULAR guidelines, patients with RA should initiate treatment with conventional synthetic disease-modifying antirheumatic drugs (csDMARDs), such as methotrexate (MTX). In patients with an inadequate response to csDMARDs, it is recommended to add a biologic DMARD (bDMARD), such as a tumor necrosis factor inhibitor (TNFi), or a targeted synthetic DMARD, such as a Janus kinase (JAK) inhibitor [5, 6].

Patients with RA who have an inadequate response to prior DMARDs have been shown to have a poorer response to subsequent treatment. In a post hoc analysis of patients with RA receiving the TNFi adalimumab (ADA) plus MTX in two randomized controlled trials, a greater number of prior csDMARDs (> 2 vs 0–1) were associated with less improvement from baseline in Disease Activity Score in 28 joints, C-reactive protein (DAS28-4[CRP]) and Health Assessment Questionnaire-Disability Index (HAQ-DI), and lower ACR response rates at week 24 [7].

Tofacitinib is an oral JAK inhibitor for the treatment of RA. The phase IIIb/IV ORAL Strategy study (NCT02187055) compared the efficacy and safety of tofacitinib 5 mg twice daily (BID) monotherapy, tofacitinib 5 mg BID + MTX, and ADA 40 mg every other week + MTX in patients with RA and an inadequate response to MTX [8]. Tofacitinib 5 mg BID + MTX demonstrated non-inferiority vs ADA + MTX, based on ACR ≥ 50% response rate (ACR50) at month 6; non-inferiority of tofacitinib 5 mg BID monotherapy vs tofacitinib 5 mg BID + MTX or ADA + MTX was not demonstrated. The mechanism by which tofacitinib + MTX provides greater efficacy than tofacitinib monotherapy is currently unknown, but is conceivably related to the effects each drug has on different mediators of inflammation.

The objective of this post hoc analysis was to further assess the differences and similarities in clinical and functional responses among treatment groups in ORAL Strategy, looking in part at patient-level data using cumulative probability plots, and evaluate the relationship among clinical efficacy measures, to guide future studies on the link between clinical efficacy measurements, or paraclinical measurements, and treatment responses.

## Methods

### Study design

ORAL Strategy (NCT02187055) was a 12-month, triple-dummy, phase IIIb/IV, active comparator, head-to-head, randomized controlled study. The full study design has been reported previously [8]. All procedures were in accordance with the Declaration of Helsinki and International Conference on Harmonization Good Clinical Practice Guidelines, and were approved by the Institutional Review Board/Ethics Committee at each study center. All patients provided written informed consent. The study was sponsored by Pfizer Inc.

### Patients

Full inclusion and exclusion criteria have been previously published [8]. Briefly, eligible patients were ≥ 18 years of age with active RA per ACR/EULAR criteria [9], despite receiving MTX for ≥ 4 months before screening and at stable doses of 15–25 mg/week (< 15 mg/week permitted only for safety reasons) for ≥ 6 weeks before baseline.

### Randomization and treatment

Patients were blindly randomized 1:1:1 to receive tofacitinib 5 mg BID (“tofacitinib monotherapy,” discontinuing MTX at the randomization visit), tofacitinib 5 mg BID + MTX (“tofacitinib + MTX”), or ADA 40 mg every other week + MTX (“ADA + MTX”).

Concomitant oral glucocorticoids (stable doses of ≤ 10 mg/day prednisone or equivalent) were permitted if a stable dose had been received for ≥ 4 weeks before the first study dose. Patients were required to discontinue all csDMARDs other than MTX for ≥ 4 weeks or 5 half-lives, whichever was longer, before baseline. Pre-study stable MTX dose was continued in patients receiving combination therapy.

### Post hoc analysis of clinical and functional responses and inflammation

Here, we assessed differences and similarities in clinical and functional responses among treatment groups in ORAL Strategy. Clinical response was primarily assessed in terms of the Clinical Disease Activity Index (CDAI), as a sensitive measure of change and a purely clinical index (i.e., not including an acute-phase reactant or physical function) [10]. Outcomes assessed were mean percent change from baseline (%∆) in CDAI at month 12, proportion of patients who achieved a ≥ 85% decrease from baseline in CDAI (previously defined as a “major response”) [11], CDAI area under the curve (AUC) at month 12, CDAI remission (≤ 2.8) and LDA (> 2.8 to ≤ 10) [10] at months 6 and 12, time-averaged (TA)-CDAI (CDAI AUC divided by 12) remission/LDA status (≤ 10), and moderate/high disease activity status (> 10) [10].

Additionally, clinical response was assessed by ACR-N (ACR response rate, where ACR is the percentage improvement from baseline in ACR components, and N represents the minimum percentage achieved by each patient—see definition below) and ACR-N AUC at month 12. ACR-N was defined as the minimum percent improvement from baseline achieved by each patient, determined by three values: %∆28-tender joint count, %∆28-swollen joint count, and median %∆ for the remaining five ACR components (Patient Global Assessment of Disease Activity, Physician Global Assessment of Disease Activity, Patient Pain, HAQ-DI, and CRP).

Functional response was assessed at month 12 by mean ∆HAQ-DI, normalized HAQ-DI (< 0.5) [12, 13], and HAQ-DI minimum clinically important difference (MCID; decrease from baseline of ≥ 0.22) [14].

Laboratory markers of inflammation were assessed in terms of median CRP and erythrocyte sedimentation rate (ESR) levels by time period (baseline, > 0–12 months, > 0–3 months, > 0–6 months, > 6–12 months). For post-baseline time periods, mean values across the time period were calculated for each patient, and the median of these mean values was then calculated across all patients.

### Statistical analyses

In order to show the distribution of changes for the population as a whole, cumulative probability plots were produced at month 12 for mean %∆CDAI, ACR-N, and mean ∆HAQ-DI.

The proportion of patients achieving normalized HAQ-DI or HAQ-DI MCID at month 12 were stratified by clinical response: CDAI remission and LDA status at months 6 and 12, and TA-CDAI remission/LDA or TA-CDAI moderate/high disease activity status.

Median CRP by time period was summarized by CDAI remission status at months 6 and 12, normalized HAQ-DI status at month 12, and HAQ-DI MCID status at month 12. Median ESR by time period was summarized by CDAI remission status at months 6 and 12.

Patients were defined as having a greater or lesser clinical or functional response per the cutoffs in Table 1.

**Table 1:** Cutoffs for greater or lesser clinical or functional response

	**Greater response**	**Lesser response**
**Clinical**		
CDAI [10]	Remission (≤ 2.8) or LDA (> 2.8 to ≤ 10) TA remission/LDA (≤ 10)	Moderate/high disease activity (> 10) TA moderate/high disease activity (> 10)
%∆CDAI [11]	≥ 85% decrease	Increase, or < 85% decrease
ACR-N [11]	≥ ACR70	< ACR70
**Functional**		
∆HAQ-DI [14]	Decrease ≥ 0.22 (MCID)	Increase, or decrease < 0.22 (non-MCID)
HAQ-DI [12, 13]	Normalized (< 0.5)	Not normalized (≥ 0.5)

*∆*, change from baseline; *ACR70*, American College of Rheumatology ≥ 70% response rate; *ACR-N*, American College of Rheumatology response rate, where ACR is the percentage improvement from baseline in American College of Rheumatology components, and N represents the minimum percentage achieved by each patient; *CDAI*, Clinical Disease Activity Index; *HAQ-DI*, Health Assessment Questionnaire-Disability Index; *LDA*, low disease activity; *MCID*, minimum clinically important difference; *TA*, time-averaged

All statistical comparisons in this analysis were considered exploratory, with no adjustment for multiplicity.

All data were summarized by treatment group. Only patients with month 12 data for ≥ 1 efficacy endpoint were included in the analyses. Observed data were used; missing values were not imputed. Patients with baseline and month 12 data were included in AUC calculations. AUC was calculated for CDAI and ACR-N (in months) with data from visits at baseline, week 6, and months 3, 6, 9, and 12, using the trapezoidal rule.

Treatment comparisons for CDAI AUC and ACR-N AUC were evaluated using analysis of variance with fixed effects of treatment and geographical region. A significance level of 0.05 was used for statistical testing. There was no adjustment for multiplicity.

## Results

### Patients

In this post hoc analysis, clinical and functional efficacy data were evaluated in the 1146 patients who received study treatment (tofacitinib monotherapy, *n* = 384; tofacitinib + MTX, *n* = 376; ADA + MTX, *n* = 386) in ORAL Strategy [8]. Prior to randomization, mean (standard deviation) MTX dose was 16.6 (3.4) mg/week in the tofacitinib monotherapy group, 16.7 (3.7) mg/week in the tofacitinib + MTX group, and 16.5 (3.7) mg/week in the ADA + MTX group.

### Clinical and functional response at month 12

At month 12, mean %∆CDAI, ACR-N, and ∆HAQ-DI were similar between the combination therapies, but numerically lower (i.e., worse response) with tofacitinib monotherapy (Table 2). Least squares mean (LSM) values for CDAI AUC for months 0 − 12 were similar between the combination therapies, but numerically lower (i.e., better response) for tofacitinib + MTX vs tofacitinib monotherapy (0.05 < *P* < 0.1), and significantly (*P* < 0.05) lower for ADA + MTX vs tofacitinib monotherapy (Table 3). Likewise, LSM values for ACR-N AUC for months 0 − 12 were similar between the combination therapies, but significantly higher (i.e., better response) for the combination therapies vs tofacitinib monotherapy (Table 3).

**Table 2:** Descriptive statistics for %∆CDAI, ACR-N, and ∆HAQ-DI at month 12

	**CDAI**			**ACR-N**		**HAQ-DI**		
	***N***^**a**^	**Baseline,** **mean (SE)**	**%∆ at month 12,** **mean (SE)**	***N***^**a**^	**At month 12,** **mean (SE)**	***N***^**a**^	**Baseline,** **mean (SE)**	**∆ at month 12,** **mean (SE)**
**Tofacitinib monotherapy**	319	38.7 (0.7)	− 65.3 (1.8)	320	32.2 (6.0)	320	1.59 (0.04)	− 0.63 (0.04)
**Tofacitinib + MTX**	309	40.5 (0.7)	− 74.0 (1.2)	310	49.1 (2.1)	311	1.57 (0.04)	− 0.67 (0.04)
**ADA + MTX**	312	38.3 (0.8)	− 72.2 (1.4)	314	45.1 (2.8)	313	1.55 (0.04)	− 0.67 (0.03)

*∆*, change from baseline; *ACR-N*, American College of Rheumatology response rate, where ACR is the percentage improvement from baseline in American College of Rheumatology components, and N represents the minimum percentage achieved by each patient; *ADA*, adalimumab; *CDAI*, Clinical Disease Activity Index; *HAQ-DI*, Health Assessment Questionnaire-Disability Index; *MTX*, methotrexate; *SE*, standard error

^a^Number of patients with month 12 data for ≥ 1 efficacy endpoint

**Table 3:** Statistical analysis of CDAI AUC and ACR-N AUC for months 0 − 12

Treatment group					Tofacitinib + MTX vs tofacitinib monotherapy^a^				Tofacitinib + MTX vs ADA + MTX^a^			
	*N*^b^	LSM	SE	95% CI	**LSM**^**c**^	**SE**	**95% CI**	***P* value**	**LSM**^**d**^	**SE**	**95% CI**	***P* value**
**CDAI AUC**^**e**^ for months 0 − 12												
Tofacitinib monotherapy	319	194.9	7.1	181.0, 208.7								
Tofacitinib + MTX	309	180.0	7.0	166.3, 193.7	− 14.9	8.5	− 31.5, 1.7	0.0788	5.4	8.5	− 11.2, 22.1	0.5234
ADA + MTX	312	174.6	7.1	160.7, 188.4	− 20.3	8.4	− 36.8, − 3.8	0.0162*				
**ACR-N AUC**^**e**^for months 0 − 12												
Tofacitinib monotherapy	320	318.7	35.3	249.3, 388.1								
Tofacitinib + MTX	310	436.8	35.0	368.1, 505.4	118.1	42.6	34.4, 201.7	0.0057**	34.4	42.8	− 49.6, 118.4	0.4214
ADA + MTX	314	402.3	35.4	332.9, 471.8	83.7	42.5	0.3, 167.0	0.0491*				

*ACR-N*, American College of Rheumatology response rate, where ACR is the percentage improvement from baseline in American College of Rheumatology components, and N represents the minimum percentage achieved by each patient; *ADA*, adalimumab; *AUC*, area under the curve; *CDAI*, Clinical Disease Activity Index; *CI*, confidence interval; *LSM*, least squares mean; *MTX*, methotrexate; *SE*, standard error

*P < 0.05, **P < 0.01, combination treatment vs tofacitinib monotherapy

^a^Treatment comparisons were analyzed using analysis of variance at month 12 with fixed effects of treatment and geographic region (North America; Latin America; Eastern Europe, Israel, and South Africa; Western Europe and Turkey; Asia and Australia)

^b^Number of patients with month 12 data for ≥ 1 efficacy endpoint

^c^Combination treatment minus tofacitinib monotherapy

^d^Tofacitinib + MTX minus ADA + MTX

^e^Calculated in months

### Cumulative probability plots: clinical and functional response at month 12

**Fig. 1:**
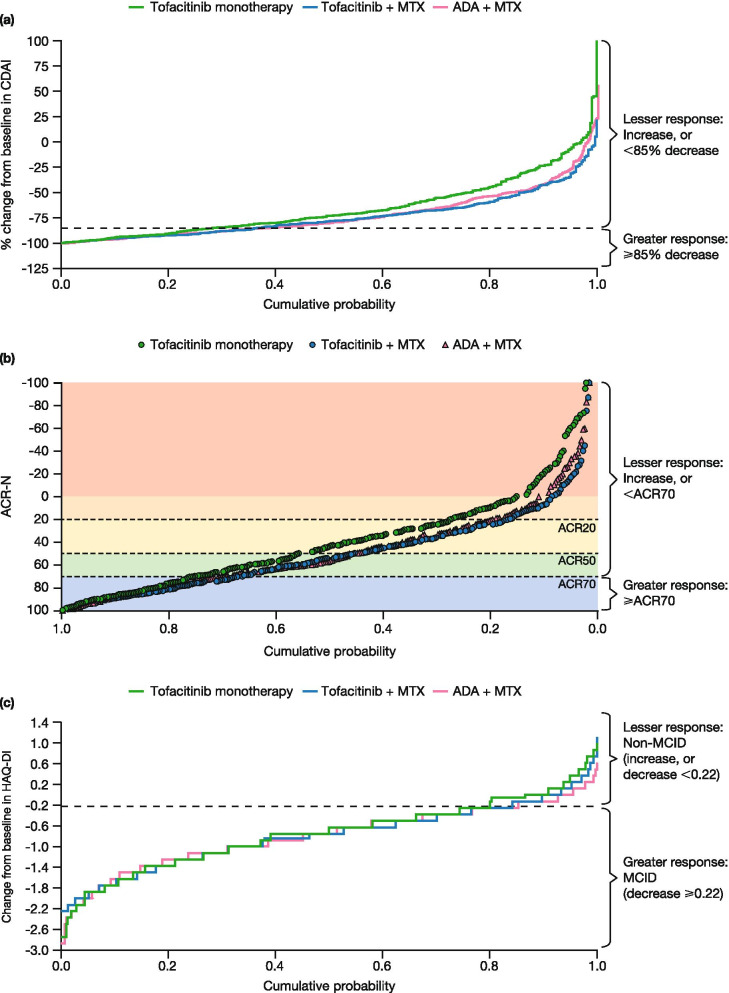
Cumulative probability plots at month 12. Plots show the cumulative probability for **a** %∆CDAI^a^, **b** ACR-N^b^, and **c** ∆HAQ-DI. ^a^One extreme value for %∆CDAI is not shown: tofacitinib monotherapy, %∆CDAI = 175%. ^b^Ten extreme values for ACR-N are not shown: tofacitinib monotherapy, ACR-N =  − 1700, − 200, − 167, − 160, and − 150; cumulative probability = 0.003, 0.006, 0.009, 0.013, 0.019; tofacitinib + MTX, ACR-N =  − 106, cumulative probability = 0.003; ADA + MTX, ACR-N =  − 463, − 167, − 158, and − 129; cumulative probability = 0.003, 0.006, 0.010, and 0.013. ∆, change from baseline; *ACR20/50/70*, American College of Rheumatology ≥ 20%, ≥ 50%, or ≥ 70% response rates; *ACR-N*, American College of Rheumatology response rate, where ACR is the percentage improvement from baseline in American College of Rheumatology components, and N represents the minimum percentage achieved by each patient; *ADA*, adalimumab; *CDAI*, Clinical Disease Activity Index; *HAQ-DI*, Health Assessment Questionnaire-Disability Index; *MCID*, minimum clinically important difference; *MTX*, methotrexate

In cumulative probability plots, at lesser levels of CDAI response (%∆CDAI increase or < 85% decrease), patients receiving tofacitinib monotherapy had a numerically poorer response than patients receiving combination therapies (Fig. 1a). The proportion of patients who achieved a greater level of response (%∆CDAI decrease ≥ 85%) was 28.8% with tofacitinib monotherapy, 37.2% with tofacitinib + MTX, and 39.1% with ADA + MTX. At this greater level of response (%∆CDAI decrease ≥ 85%), responses were similar across treatment groups in cumulative probability plots (Fig. 1a). Similar trends were observed with ACR-N (Fig. 1b) and ∆HAQ-DI (Fig. 1c).

### Association between HAQ-DI response and CDAI disease activity level

**Fig. 2:**
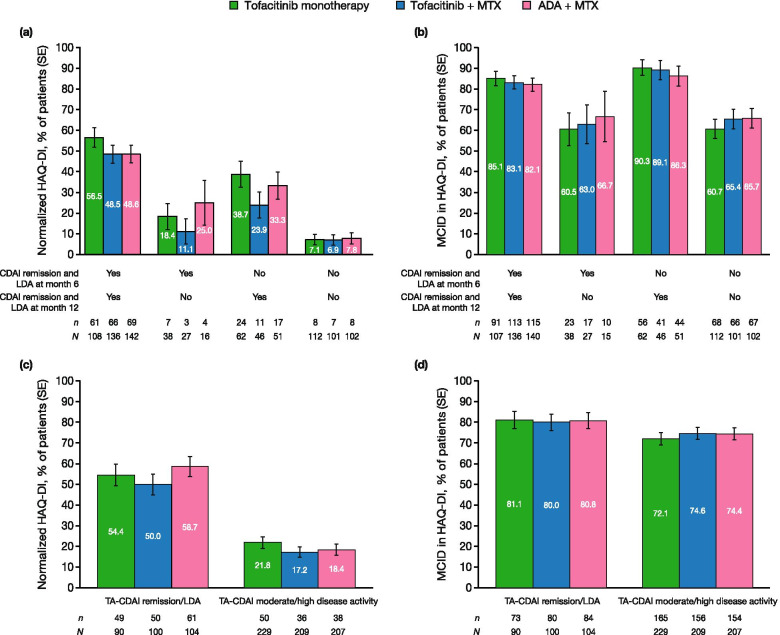
Patients with normalized HAQ-DI scores/HAQ-DI MCID at month 12, categorized by CDAI remission and LDA. Plots show **a** patients with normalized HAQ-DI scores (< 0.5) at month 12 and **b** patients achieving HAQ-DI MCID (decrease from baseline of ≥ 0.22) at month 12, categorized by CDAI remission/LDA (≤ 10) status at months 6 and 12, and **c** patients with normalized HAQ-DI scores (< 0.5) at month 12 and **d** patients achieving HAQ-DI MCID (decrease from baseline of ≥ 0.22) at month 12, categorized by TA-CDAI remission/LDA and moderate/high disease activity status. TA-CDAI was defined as CDAI AUC divided by 12. *ADA*, adalimumab; *AUC*, area under the curve; *CDAI*, Clinical Disease Activity Index; *HAQ-DI*, Health Assessment Questionnaire-Disability Index; *LDA*, low disease activity; *MCID*, minimum clinically important difference; *MTX*, methotrexate; *TA*, time-averaged; *SE*, standard error

Across treatment groups, patients with a greater clinical response at months 6 and 12 (those achieving CDAI remission/LDA) were more likely to achieve a greater functional response at month 12 (normalized HAQ-DI) than patients with a lesser clinical response (those not achieving CDAI remission/LDA) at months 6 and 12 (Fig. 2a). Similar trends were seen when greater functional response was assessed in terms of achievement of HAQ-DI MCID at month 12 (Fig. 2b), and when greater CDAI response was assessed in terms of achievement of TA-CDAI remission/LDA (Fig. 2c–d). Thus, in this group of patients, a greater improvement in CDAI was associated with greater functional improvement, as assessed by the HAQ-DI.

### Association between laboratory markers of inflammation and clinical and functional response

**Fig. 3:**
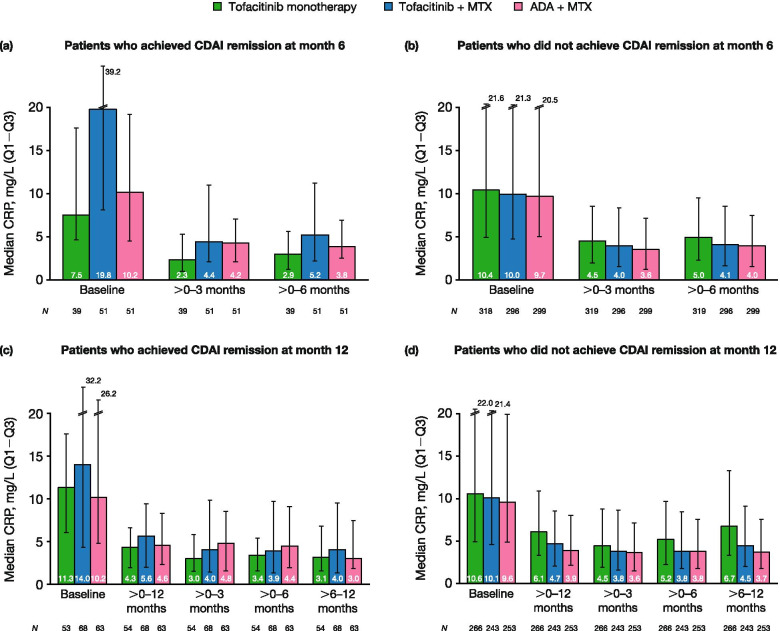
Median CRP levels by time period, categorized by CDAI remission at months 6 and 12. Plots show median CRP levels by time period for **a** patients who achieved CDAI remission (≤ 2.8) at month 6, **b** patients who did not achieve CDAI remission (> 2.8) at month 6, **c** patients who achieved CDAI remission (≤ 2.8) at month 12, and **d** patients who did not achieve CDAI remission (> 2.8) at month 12. Error bars represent the interquartile range (Q1‒Q3). *ADA*, adalimumab, *CDAI*, Clinical Disease Activity Index; *CRP*, C-reactive protein; *MTX*, methotrexate; *Q1*, 25th percentile; *Q3*, 75th percentile

**Fig. 4:**
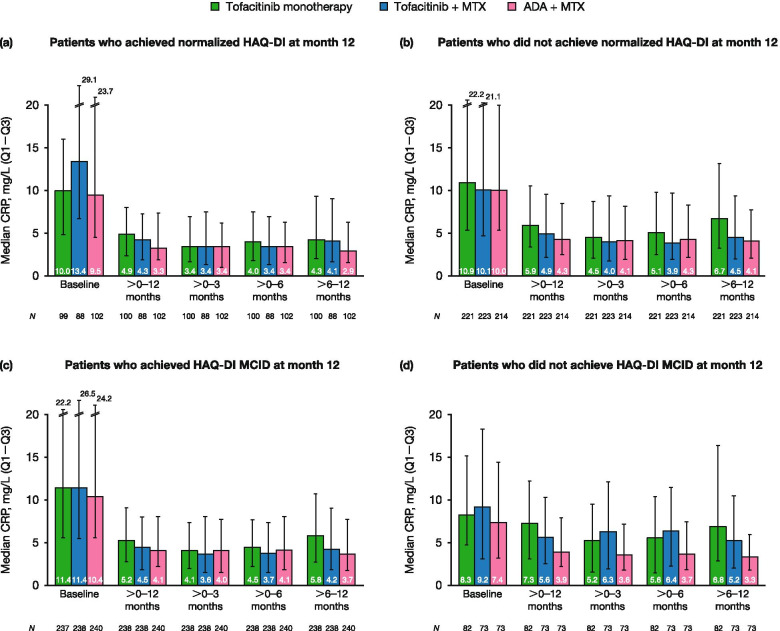
Median CRP levels by time period, categorized by HAQ-DI status at month 12. Plots show the median CRP levels for **a** patients who achieved normalized HAQ-DI (< 0.5) at month 12, **b** patients who did not achieve normalized HAQ-DI (≥ 0.5) at month 12, **c** patients who achieved HAQ-DI MCID (decrease from baseline of ≥ 0.22) at month 12, and **d** patients who did not achieve HAQ-DI MCID (increase, or decrease < 0.22 from baseline) at month 12. Error bars represent the interquartile range (Q1–Q3). *ADA*, adalimumab; *CRP*, C-reactive protein; *HAQ-DI*, Health Assessment Questionnaire-Disability Index; *MCID*, minimum clinically important difference; *MTX*, methotrexate; *Q1*, 25th percentile; *Q3*, 75th percentile

Patients receiving tofacitinib + MTX with greater clinical response at month 6 (Fig. 3a) or month 12 (Fig. 3c), and greater functional response at month 12 (Fig. 4a and c), had numerically higher median baseline CRP levels and larger post-baseline CRP reductions, compared with patients with a lesser clinical (Fig. 3b, d) and functional (Fig. 4b, d) response. However, interquartile ranges were wide and overlapping. There was not such a clear trend in the tofacitinib monotherapy and ADA + MTX groups; again, interquartile ranges were wide and overlapping.

Findings were similar when inflammation was assessed by median ESR levels in patients with greater vs lesser clinical response at month 6 (see Additional file 1, Figure S1a and b) and month 12 (see Additional file 1, Figure S1c and d).

## Discussion

In this post hoc analysis, we assessed clinical and functional responses between treatment groups in ORAL Strategy in greater depth than previously reported [8]. Our results support the primary findings of ORAL Strategy, which demonstrated non-inferiority of tofacitinib + MTX vs ADA + MTX, based on ACR50 response rates at month 6, while non-inferiority of tofacitinib monotherapy vs tofacitinib + MTX was not demonstrated [8]. In our analysis, clinical and functional responses at month 12 were generally similar with tofacitinib + MTX and ADA + MTX, but somewhat lower with tofacitinib monotherapy. Using cumulative probability plots, we showed that clinical and functional responses assessed across the study population were similar among the three treatment groups at greater levels of response. At lower levels of response, however, patients receiving tofacitinib monotherapy vs combination therapies did not appear to respond as well.

Overall, these results suggest that in a group of patients with RA, tofacitinib + MTX may provide greater efficacy than tofacitinib monotherapy, but the reason for these differences is not fully understood. Multiple cytokines are involved in the pathology of RA, and it is hypothesized that treatment may result in changes in dominant pathological cytokines [15], and modification of gene transcription, protein levels, and immunophenotype that are aligned with altered disease activity [16]. It has previously been shown that in patients with RA, clinical and functional responses are greater with bDMARDs in combination with MTX, compared with bDMARD monotherapy [17–20]. While bDMARDs target extracellular elements of the inflammation pathway, JAK inhibitors directly bind to, and modulate, the intracellular catalytic activity of JAKs, which are essential enzymes in the JAK/signal transducer and activator of transcription (STAT) signaling pathways of type I and type II cell-surface cytokine receptors [21–24]. It is possible that concomitant MTX may suppress cytokines, such as interleukin (IL)-1 [25], IL-17 [26, 27], and TNF [28], which do not signal via the JAK/STAT pathway [24]. MTX may additionally exhibit a synergistic effect with bDMARDs, due to its effect in inhibiting the production of neutralizing antibodies against the bDMARD [29, 30]. Tofacitinib is a small molecule [22] and therefore unlikely to induce the formation of neutralizing anti-drug antibodies. The effects of JAK inhibitors, including tofacitinib, on the immunogenomic network have been investigated in in vitro and murine models [29–31], as well as in a phase II synovial biopsy study, in which tofacitinib reduced expression of genes implicated in RA, including matrix metalloproteinase and interferon-regulated chemokines [32].

Additionally, we found that across treatment groups, reaching the clinical response target of CDAI remission/LDA at month 12 was associated with improvement in functional response (achievement of normalized HAQ-DI or HAQ-DI MCID) at month 12. It has previously been reported that there is a direct association between increased disease activity and reduced functional capacity [33–36]. These findings support the hypothesis that treating RA with a target of remission or LDA allows many patients to reach normative functional capacity.

To improve our understanding of the differences we observed in clinical and functional responses, we analyzed laboratory markers of inflammation. With tofacitinib + MTX, numerically higher baseline CRP levels, and numerically larger post-baseline CRP reductions, were seen in patients achieving greater vs lesser clinical and functional response at months 6 and 12. These results suggest that high pre-treatment CRP levels and early post-baseline CRP reductions may be associated with better response to tofacitinib + MTX. However, interquartile ranges were wide and overlapping, and no formal statistical comparisons were conducted, so results should be interpreted with caution.

Limitations of this analysis must be considered. This was a post hoc analysis from a single study not designed to investigate the objectives of this analysis. Additionally, this analysis included only patients with complete data; missing values were not imputed. Loss of patients over time could have led to attrition biases which overestimated treatment response. Furthermore, the size of some subgroups was small, and MCID data were only evaluated at limited time points. The number of analyses carried out could have increased the risk of a type I error; all comparisons were considered exploratory. Also, this analysis did not assess the impact of some factors which can affect response, such as smoking status, seropositivity (for rheumatoid factor and/or anti-citrullinated protein antibodies), or duration of RA. In addition, assessment of ACR-N AUCs can be prone to error, as small variations in ACR-N AUC at study baseline can be inflated over time and lead to possible interpretation bias [37]. Finally, serum IL-6 was not measured, and measurement of functional capacity was limited to HAQ-DI rather than including other patient-reported outcomes.

## Conclusions

In this post hoc analysis of data from ORAL Strategy, cumulative probability plots suggest that there is a subset of patients who will achieve a deep clinical and functional response with tofacitinib treatment, irrespective of whether they receive concomitant MTX. However, at lower levels of response, patients receiving tofacitinib monotherapy did not appear to respond as well as those receiving combination treatments. Overall, patients experienced greater response with tofacitinib + MTX vs tofacitinib monotherapy. As such, tofacitinib monotherapy may be considered in patients who wish to discontinue MTX due to tolerability or toxicity issues. However, in patients who are able to take MTX, as recommended by ACR and EULAR, tofacitinib should be added to MTX, and tapering or discontinuation of MTX should be considered only after the patient reaches their targeted disease control [5, 6].

## Supplementary Information

**Additional file 1:Figure S1. **Median ESR levels by time period. Median ESR levels by time period for patients who: achieved CDAI remission (≤2.8) at month 6; did not achieve CDAI remission (>2.8) at month 6; achieved CDAI remission (≤2.8) at month 12; and did not achieve CDAI remission (>2.8) at month 12

## Data Availability

Upon request, and subject to certain criteria, conditions, and exceptions (see https://www.pfizer.com/science/clinical-trials/trial-data-and-results for more information), Pfizer will provide access to individual de-identified participant data from Pfizer-sponsored global interventional clinical studies conducted for medicines, vaccines, and medical devices (1) for indications that have been approved in the US and/or EU, or (2) in programs that have been terminated (i.e., development for all indications has been discontinued). Pfizer will also consider requests for the protocol, data dictionary, and statistical analysis plan. Data may be requested from Pfizer trials 24 months after study completion. The de-identified participant data will be made available to researchers whose proposals meet the research criteria and other conditions, and for which an exception does not apply, via a secure portal. To gain access, data requestors must enter into a data access agreement with Pfizer.

## Abbreviations

ACR: American College of Rheumatology
ACR20/50/70: American College of Rheumatology ≥ 20%, ≥ 50%, or ≥ 70% response rates
ACR-N: American College of Rheumatology response rate, where ACR is the percentage improvement from baseline in American College of Rheumatology components, and N represents the minimum percentage achieved by each patient
ADA: Adalimumab
AUC: Area under the curve
bDMARD: Biologic disease-modifying antirheumatic drug
BID: Twice daily
CDAI: Clinical Disease Activity Index
CI: Confidence interval
CRP: C-reactive protein
csDMARD: Conventional synthetic disease-modifying antirheumatic drug
DAS28-4(CRP): Disease Activity Score in 28 joints, C-reactive protein
DMARD: Disease-modifying antirheumatic drug
ESR: Erythrocyte sedimentation rate
EULAR: European Alliance of Associations for Rheumatology
HAQ-DI: Health Assessment Questionnaire-Disability Index
IL: Interleukin
JAK: Janus kinase
LDA: Low disease activity
LSM: Least squares mean
MCID: Minimum clinically important difference
MTX: Methotrexate
Q1: 25th percentile
Q3: 75th percentile
RA: Rheumatoid arthritis
SD: Standard deviation
SE: Standard error
STAT: Signal transducer and activator of transcription
TA: Time-averaged
TNF: Tumor necrosis factor
TNFi: Tumor necrosis factor inhibitor
