# Supplementary material for: Differences and similarities in clinical and functional responses among patients receiving tofacitinib monotherapy, tofacitinib plus methotrexate, and adalimumab plus methotrexate: a post hoc analysis of data from ORAL Strategy
Source: Arthritis Res Ther. 2021 Aug 24;23:220. doi: 10.1186/s13075-021-02591-y (PMC8383419; doi:10.1186/s13075-021-02591-y)
Supplement: Supplementary file 1 — Additional file 1:Figure S1. Median ESR levels by time period. Median ESR levels by time period for patients who: achieved CDAI remission (≤2.8) at month 6; did not achieve CDAI remission (>2.8) at month 6; achieved CDAI remission (≤2.8) at month 12; and did not achieve CDAI remission (>2.8) at month 12 [file 13075_2021_2591_MOESM1_ESM.pdf]

Additional file 1

Fig. S1 Median ESR levels by time period, categorized by CDAI remission status at months 6 and 12

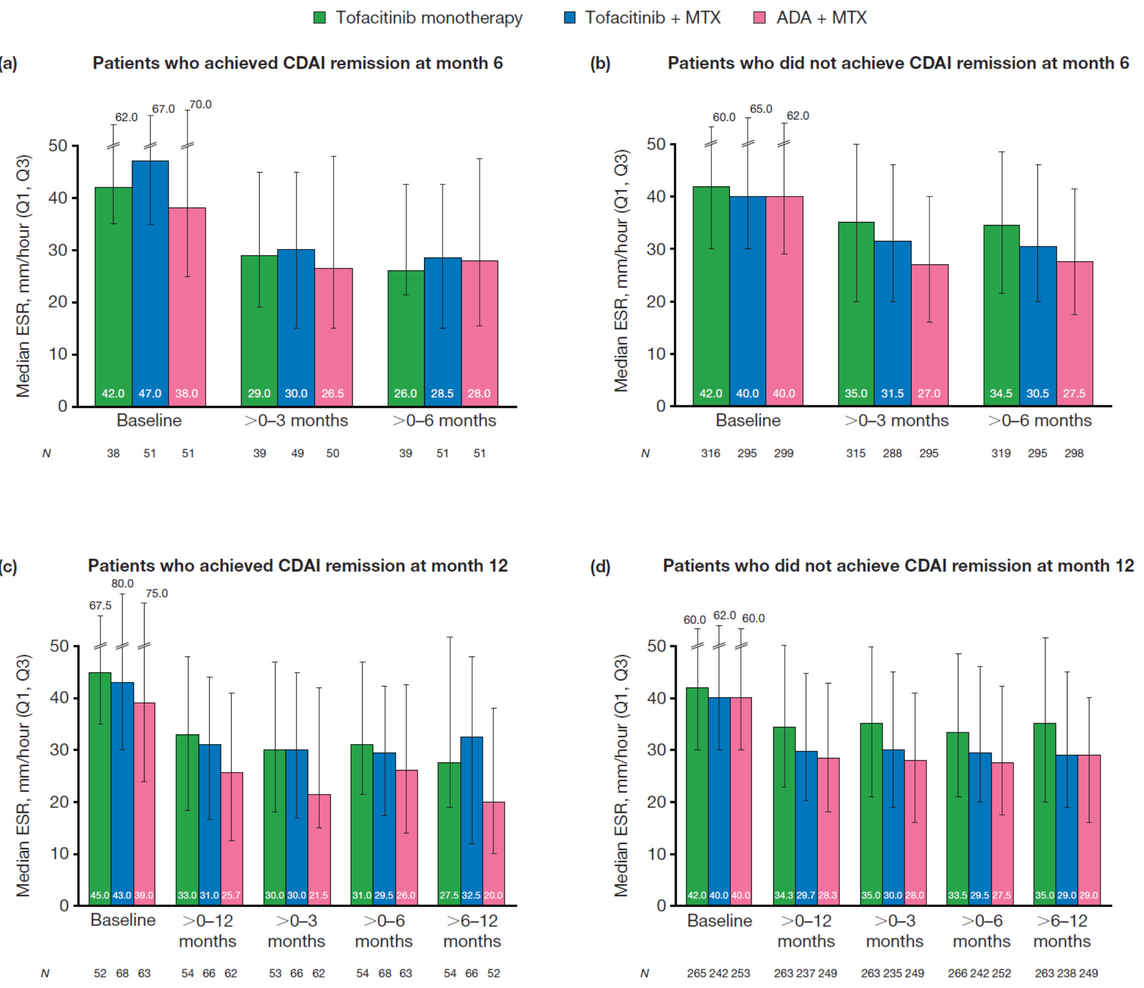

Plots show median ESR levels by time period for (a) patients who achieved CDAI remission (≤2.8) at month 6, (b) patients who did not achieve CDAI remission (>2.8) at month 6, (c) patients who achieved CDAI remission (≤2.8) at month 12, and (d) patients who did not achieve CDAI remission (>2.8) at month 12. Error bars represent the interquartile range (Q1–Q3). *ADA* adalimumab, *CDAI* Clinical Disease Activity Index, *ESR* erythrocyte sedimentation rate, *MTX* methotrexate, *Q1* 25th percentile, *Q3* 75th percentile
